# Causal inference of the effect of blood proteome on the risk of head and neck cancer: two-sample Mendelian randomization

**DOI:** 10.1007/s12672-024-01128-4

**Published:** 2024-07-10

**Authors:** Zhen Wang, Jianhao Wu

**Affiliations:** Department of Stomatology, The Quzhou Affiliated Hospital of Wenzhou Medical University (Quzhou People’s Hospital), Kecheng District, Minjiang Avenue No. 100, Quzhou, 332400 Zhejiang Province China

## Abstract

Early diagnosis of head and neck cancer can improve therapeutic outcomes but remains a challenge. The blood proteome can comprise a key source of biomarkers that enable the early diagnosis and precision medicine in head and neck cancer, but blood protein biomarkers of head and neck cancer are not well delineated. Here we applied two-sample Mendelian randomization to a GWAS dataset of 1478 blood proteins and large dataset of head and neck cancer cases and controls to identify blood proteome traits associated with head and neck cancer. Multiple two-sample Mendelian randomization (MR) methods were used to assess causal effects of the exposures, including: Inverse-variance weighted (IVW), Mendelian randomization-Egger method, Weight Median method, simple mode, weight mode. Sensitivity analysis was performed by using heterogeneity test, pleiotropy test and one-by-one exclusion test. Multivariable MR analyses were performed to assess the effects of obesity, diabetes mellitus, and smoking. A significant causal association between A Disintegrin and metalloproteinase domain-containing protein 23 (ADAM23) and head and neck cancer was noted. The sensitivity analysis indicated no significant bias. Multivariate analysis showed that the effect for ADAM23 remained significant after adjusting for the indirect effects of obesity, diabetes mellitus and smoking. In sum, this study showed a significant causal role of genetically dysregulated ADAM23 protein with head and neck cancer risk. The specific mechanisms underlying the role of ADAM23 in mediating head and neck cancer risk, and its role as a potential therapeutic target and biomarker, need further investigation.

## Introduction

Head and neck cancer imposes significant and increasing global public health burden, with particularly higher burden in low- and middle-income countries [1]. Globally, more than 900,000 cases of head and neck cancer are reported annually, and it comprises the 6th most common cancer type [2]. Collectively, head and neck cancers include epithelial cancers of the oral cavity, pharynx, larynx, nasal and paranasal regions [3]. Most of the head and neck cancers comprise squamous cell carcinomas [4] and the major risk factors include smoking or smokeless tobacco consumption, betel nut chewing, alcohol, and virus including Human Papillomavirus (HPV) and Epstein Barr Virus (EBV) [5, 6]. Head and neck cancers are frequently diagnosed at the late stages [6, 7] leading to worse survival outcomes [8]. Although 5-year survival rates have improved in the past decades [9], overall survival rates still typically remain below 70% [10]. The primary treatment modalities include surgery and radiotherapy, which are associated with significant morbidity and reduction in quality of life [11]. The need for expanding clinically validated biomarkers that enable early diagnosis and targeted therapies has been recognized as a priority [12].

Head and neck cancers are characterized by high mutational burden but display a complex and heterogenous molecular subtypes [13]. A deeper understanding of the mutational, genomic, and molecular landscape of head and neck cancer is essential to advance early-stage biomarker research and targeted therapeutics [13, 14]. Blood protein biomarkers are advantageous due to the minimally invasive nature of their collection and ability for repeated sampling for disease monitoring. Currently, available blood protein biomarkers for head and neck cancer include glutathione S-transferase P1 (GSTP1), cyclase-associated protein 1 (CAP1), osteopontin (OPN), and cellular fibronectin (cFN), among others. These biomarkers have shown potential in the diagnosis, prognosis, and disease monitoring of head and neck cancer. For instance, the concentration of GSTP1 is significantly lower in patients with oral squamous cell carcinoma (OSCC) compared to healthy individuals. Serum levels of CAP1 correlate with the depth of tumor invasion and the presence of regional metastases. OPN is significantly elevated in patients with laryngeal and hypopharyngeal squamous cell carcinoma. Additionally, fibronectin levels are increased in the plasma of patients with head and neck squamous cell carcinoma. However, these biomarkers also have certain limitations. For example, the expression differences of GSTP1 and CAP1 may be influenced by other non-cancerous factors. While OPN levels are elevated in tumor patients, their expression may vary with tumor differentiation and metastasis, and inconsistent results have been reported among different studies. Therefore, although these plasma protein biomarkers show potential in the diagnosis and prognosis of head and neck cancer, further validation and optimization are needed for their clinical application [15].

Blood proteome and Mendelian randomization analysis are powerful tools for uncovering novel disease biomarkers and elucidating pathogenic mechanisms. Recent advancements in proteomics technology, such as high-throughput platforms and liquid chromatography-tandem mass spectrometry (LC–MS/MS), have greatly enhanced the depth and precision of proteomic research [16]. These advancements have increased the resolution and depth of proteomics research, allowing for the discovery of protein interactions and functional pathways through the systems biology analysis, thus providing new biomarkers and therapeutic targets. Integrating genomic and proteomic data can identify key protein networks closely associated with disease, elucidating their roles in disease onset and progression [17].

The blood proteome is significantly impacted during cancer development and progression, and thereby is an important source of cancer biomarkers [18], while rapid advances in high-throughput technology now allow the capture of proteomic signatures at scale and precision [19]. Several genome wide association studies (GWAS) have documented genetic variants and single nucleotide polymorphisms (SNPs) associated with head and neck cancer [20, 21], however these studies do not clarify the underlying mechanisms and pathways at play in the translation of genetic variants to protein and phenotype level variations. GWAS analyses have also highlighted genetic variants linked to blood protein concentration (protein quantitative trait loci) and have also indicated their causal role in disease expression [22]. Complex causal relationships of genetic variants as instrument variables with outcomes can be inferred by a Mendelian randomization approach, while avoiding environmental confounders [22–24]. Additionally, integrating the knowledge of genetic variants associated with circulating protein quantities with genetic variants from GWAS studies can shed light on causal mechanisms linking genetic variants with protein-level changes [25]. A two-sample Mendelian randomization (two-sample MR) study design involves the application of Mendelian randomization methods to summary data from distinct sample sets, such as that from GWAS studies [26]. The integrated application of blood proteome and Mendelian randomization analysis can reveal key disease-associated protein changes and their upstream genetic mechanisms at multiple levels, accelerating the discovery and validation of biomarkers, elucidating pathogenic molecular pathways, and providing novel strategies and targets for disease prevention, diagnosis, and treatment [27].

To address these knowledge gaps, we employed a two-sample MR approach to comprehensively evaluate the causal effects of blood protein levels on head and neck cancer risk. By leveraging summary statistics from large-scale GWAS of blood protein levels and head and neck cancer, we aimed to identify specific proteins that are causally implicated in head and neck carcinogenesis. The use of genetic variants as instrumental variables in MR analysis allows for the assessment of causal relationships while minimizing confounding and reverse causation bias. Furthermore, we performed extensive sensitivity analyses to verify the robustness of our findings and conducted multivariable MR analysis to account for potential pleiotropic effects of genetic instruments. Through this study, we sought to provide novel insights into the etiologic role of the blood proteome in head and neck cancer development and identify promising biomarkers and therapeutic targets for this malignancy.

## Materials and methods

### Study reporting guidelines and study design

**Fig. 1:**
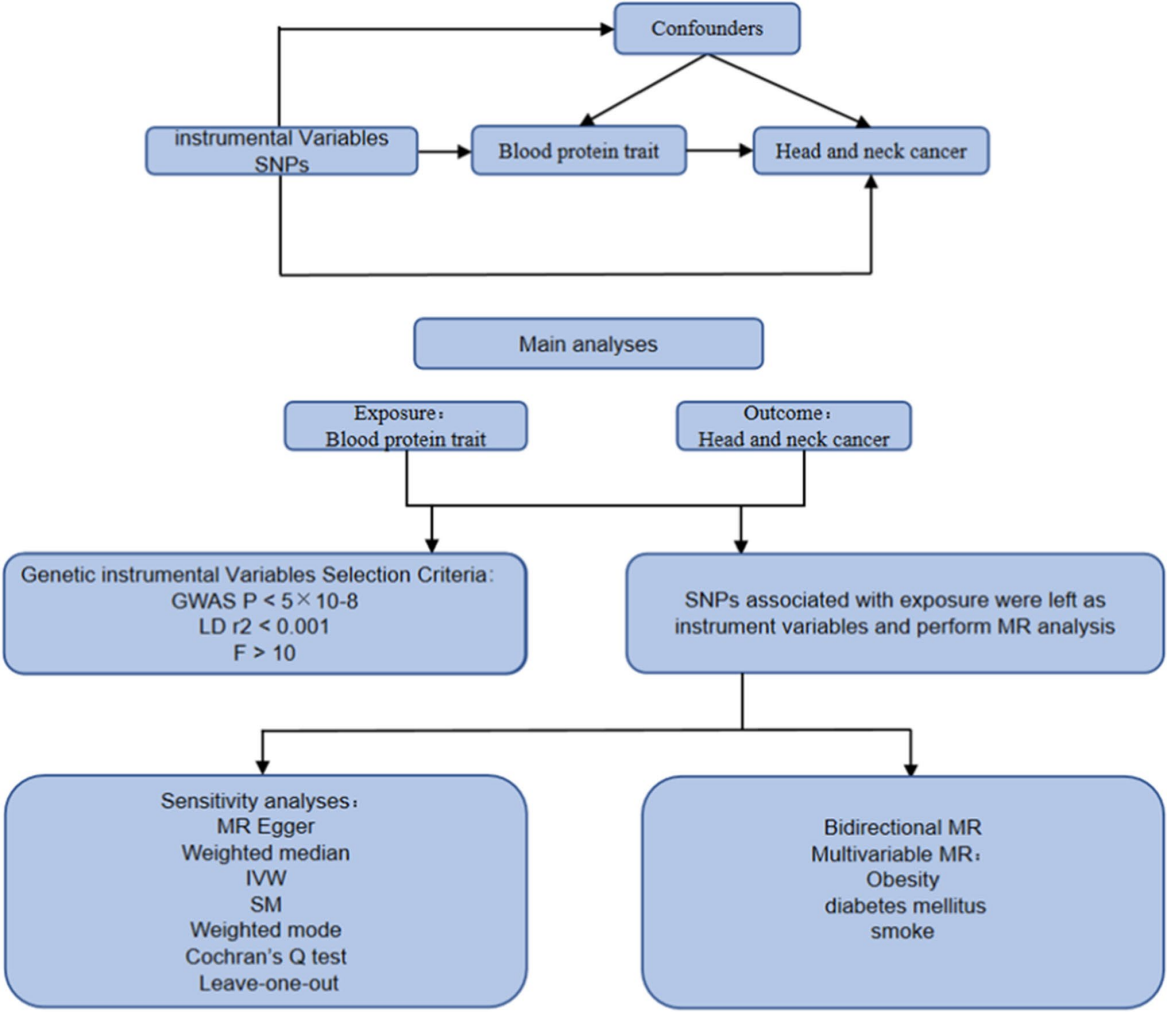
Flow of Mendelian randomization analysis of the effect of blood proteome on the risk of head and neck cancer. SNPs: Single Nucleotide Polymorphisms. F: F statistics. MR: Mendelian randomization. Q: Cochran's Q test statistic. IVW: Inverse variance weighted. SM: Simple mode

A two-sample MR approach using publicly available datasets was applied to assess the effect of blood protein traits on head and neck cancer. The study report was constructed according to the Mendelian randomization reporting guidelines by Pietzner et al. (2021) [27]. The study design diagram is shown in Fig. 1.

### Data sources

Blood proteome GWAS data included GWAS analysis of 1478 proteins studied by Sun et al. [22]. GWAS data of head and neck cancer included a large-scale multiphenotypic GWAS meta-analysis conducted by Burrows et al., including 373,122 study subjects from the UK Biobank, including 1106 cases of head and neck cancer and 372,016 controls [28]. The participant groups of these two studies do not overlap, meeting the requirement of using independent samples for the two-sample Mendelian randomization analysis, which helps avoid bias caused by the winner's curse phenomenon.

Other GWAS data include GWAS data of obesity from the GWAS analysis data of FinnGen Consortium, including 8908 cases and 209,827 controls, GWAS data of diabetes mellitus (DM) from GWAS analysis data of FinnGen Consortium, including 35,607 cases and 183,185 controls [29]. The GWAS data of smoking [30] were from the GWAS analysis data by Liu et al. in which there were 311,629 cases and 321,173 controls for smoking.

### Instrumental variable selection

A valid genetic variation instrumental variable is required to satisfy three core assumptions: (1) the hypothesis of association, that is, the selected instrumental variable must be significantly related to the exposure factor, (2) independence assumption, that is, the instrumental variable must not be significantly related to potential confounders that may affect the exposure or outcome, (3) exclusivity limitation, that is, the instrumental variable can only affect the outcome through the path of "instrumental variable → exposure → outcome".

In this study, the instrumental variable screening criteria for exposure were as follows: SNPS in linkage disequilibrium (SNPS with r^2^< 0.001 and physical distance > 10,000 kb between genes) were excluded when the P value of Single Nucleotide Polymorphism (SNP) in GWAS was less than 5 × 10^–8^. Then, instrumental variables were extracted from the GWAS of the outcome data according to the selected SNPS. F-statistics were calculated to assess weak instrumental variable bias [31]. When F < 10, it indicates that the genetic variation used is a weak instrumental variable, which may produce bias, so it should be removed. The formula for calculating the F-statistics is as follows:

$$F = \,\frac{N - K - 1}{K} * \frac{{R^{2} }}{{1 - R^{2} }}$$

where N is the sample size, K represents the number of instrumental variables used, and R^2^ reflects the extent to which the instrumental variables explain the exposure. R^2^ = 2 × (1-MAF) × MAF × ^2^β, where MAF is the minimum allele frequency and β is the allele effect size.

### MR causal effect estimation

Multiple two-sample Mendelian randomization methods were used to assess causal effects of the exposures on outcomes, including: Inverse-variance weighted (IVW), Mendelian randomization-Egger method, Weight Median method, simple mode, weight mode. It has been shown that the IVW method [32] is slightly stronger than others under some conditions. Its characteristics are that the existence of intercept term is not considered in the regression, and the inverse of the outcome variance is used as the weight for fitting. Therefore, in the absence of pleiotropy and with or without heterogeneity, the IVW method was used as the main MR Analysis, supplemented by the other four methods (IVW random effects model was used in the presence of heterogeneity). When pleiotropy was present, MR-Egger method was used to calculate the results. Reverse causality was assessed in the same way as the possible causal effect of the outcome on the exposure.

### Sensitivity analysis

Sensitivity analysis was carried out by using heterogeneity test, pleiotropy test and one-by-one exclusion test, as follows:

1. Heterogeneity test: The Cochran Q test was used to evaluate the heterogeneity among the SNP estimates. If the Cochran Q test was statistically significant, the analysis results were considered to be significantly heterogeneous. The random effects model of IVW was used to evaluate the causal effect size for highly heterogeneous results. Because the Cochran Q test can only test the presence or absence of heterogeneity and cannot test the distribution of heterogeneity, the I^2^ statistic was used to reflect the heterogeneity attributable to the instrumental variable in the total variation: when I^2^ ≤ 0, it was set to 0, indicating no observed heterogeneity. I^2^ = 0 to 25%, indicating mild heterogeneity. I^2^ = 25 to 50%, indicating moderate heterogeneity. I^2^ > 50% indicated high heterogeneity. The specific calculation formula is as follows:
  $$I^{2} = \,\frac{{Q - {\text{df}}}}{Q} * 100\%$$

(2) Pleiotropy test: MR-Egger method was used to test the pleiotropy of instrumental variables. Significant horizontal pleiotropy of genetic variation was indicated if the P value of MR-Egger's intercept was less than 0.05.
(3) Leave-one-out test: The MR Results of the remaining instrumental variables were calculated by excluding single SNP one by one to assess whether the SNP affected the association between blood proteome indicators and the risk of head and neck cancer. If there was a large difference between the MR Effect estimates and the total effect estimates after excluding an instrumental variable, it indicated that the MR Effect estimates were sensitive to that SNP.

### Statistical analysis

All data calculations and statistical analyses were programmed using R (https: //www.r-projec t.org/, version 4.3.0). The ‘TwoSampleMR’ package was mainly used for Mendelian randomization analysis [33]. The Cochran Q test and leave-one-out analysis were used to evaluate the robustness and reliability of the results. Genetic pleiotropy test was performed by MR-Egger intercept method. The evaluation indicators were odds ratio (OR) and 95% confidence interval (95% CI). All statistical P values were two-sided, and P < 5 × 10^–8^ was considered statistically significant for SNPS generated from GWAS studies. For other statistical tests, P < 0.05 was considered statistically significant, and Bonferroni correction was used for multiple testing. The threshold was set as 0.05/36 = 0.0014.

### Analysis of the clinical application potential of ADAM23 in head and neck squamous cell carcinoma

To assess the expression, diagnostic value, and prognostic significance of ADAM23 in head and neck squamous cell carcinoma (HNSC), we performed a series of analyses using public databases. First, we used the GEPIA database (http://gepia.cancer-pku.cn/) to compare the expression levels of ADAM23 in HNSC tumor tissues (n = 519) and normal tissues (including TCGA adjacent normal tissues and GTEx normal tissues, n = 44). In the GEPIA database, we selected the following parameter settings: tumor color as pink, normal tissue color as gray, |Log2FC| cutoff value set to 1, p-value cutoff set to 0.01, using log2(TPM + 1) for log transformation, and jitter size set to 0.4. The results were presented in the form of a box plot.

Second, we evaluated the diagnostic value of ADAM23 in distinguishing HNSC tumor tissues from normal tissues. We used the pROC package (version 1.18.0) in R language (version 4.2.1) for ROC curve analysis and visualized the results using the ggplot2 package (version 3.3.6). The ROC curve reflects the relationship between sensitivity and specificity, and the area under the curve (AUC) is used to evaluate the efficacy of diagnostic tests. An AUC value closer to 1 indicates better diagnostic performance. We used normal tissues as the reference group and calculated sensitivity, specificity, true positives, false positives, true negatives, and false negatives.

Finally, we used the Kaplan–Meier Plotter online tool (https://kmplot.com/analysis/) to investigate the relationship between ADAM23 expression levels and the prognosis of HNSC patients. This tool can assess the correlation between gene expression (mRNA, miRNA, protein, and DNA) and survival in over 35,000 samples from 21 tumor types. The applied statistical methods include Cox proportional hazards regression and the calculation of the false discovery rate. We used this tool to analyze the relationship between ADAM23 expression levels and overall survival (OS) and recurrence-free survival (RFS) in HNSC patients, based on RNA-seq data from 500 HNSC patients.

## Results

### Instrumental variable screening

According to the screening criteria of instrumental variables in this study, SNPS with linkage disequilibrium were removed. After matching with the GWAS data of head and neck cancer, SNPS related to blood proteome indicators were included as instrumental variables. The number of instrumental variables of each indicator is shown in Table 1. Only the indicators with significant results (P value < 0.05) in MR Analysis are shown in Table 1, and the F-test statistics of the instrumental variables of these indicators are all greater than 10, indicating that the SNPS screened in this study were strong-effect instrumental variables, and the possible bias caused by weak instrumental variables is limited.

**Table 1:** Selection of instrumental variables for blood protein trait and head and neck cancer. SNPs: Single Nucleotide Polymorphisms. F: F statistics

Exposure	Number of SNPs	Minimum of F	Median of F	Maximum of F
A Disintegrin and metalloproteinase domain—containing protein 23 | | id: prot-a-28	5	30.63258886	40.32267077	286.9623782

### MR Causal effect estimates

**Fig. 2:**
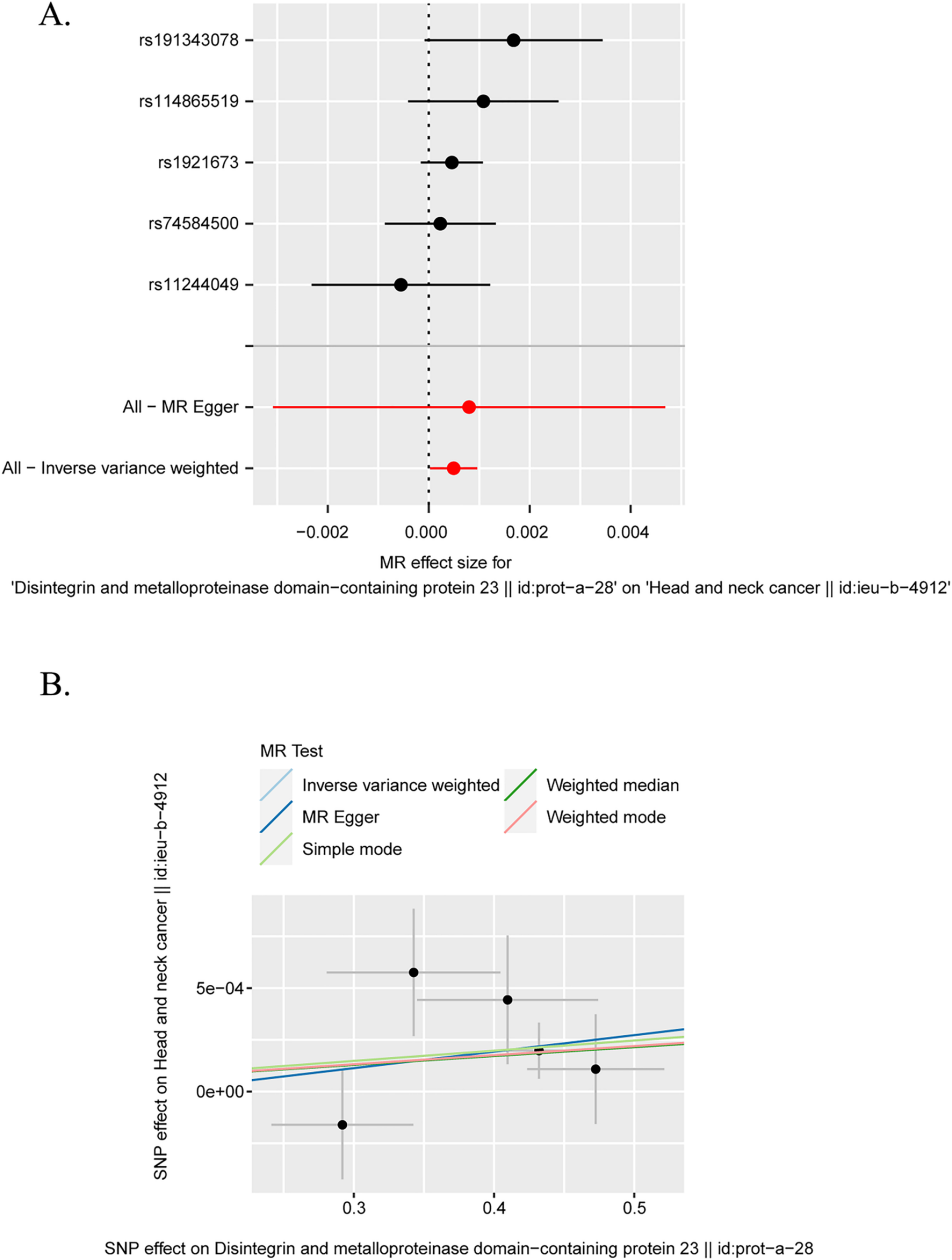
Multiple model analysis results and effect estimation of the Mendelian randomization analysis of the effect of blood proteome on the risk of head and neck cancer. **A** The results of multiple Mendelian randomization model analysis on the causal association between blood proteome indicators and the risk of head and neck cancer are presented in the forest plot.** B** Scatter plot shows the causal relationship between ADAM23 and head and neck cancer, and the slope of the line indicates the magnitude of the causal relationship predicted by different models

MR Egger, Weighted median, Inverse variance weighted (IVW), Simple mode (SM), Weighted model was each used for analysis. The results are shown in the forest plot (Fig. 2A).

IVW model results showed a significant causal association between A Disintegrin and metalloproteinase domain-containing protein 23 (ADAM23) and head and neck cancer.

Subsequently, we plotted scatter plots to show that the MR Analysis of different models of ADAM23 (Fig. 2B) gave consistent direction estimates and their slopes were consistent.

### Sensitivity analysis

**Fig. 3:**
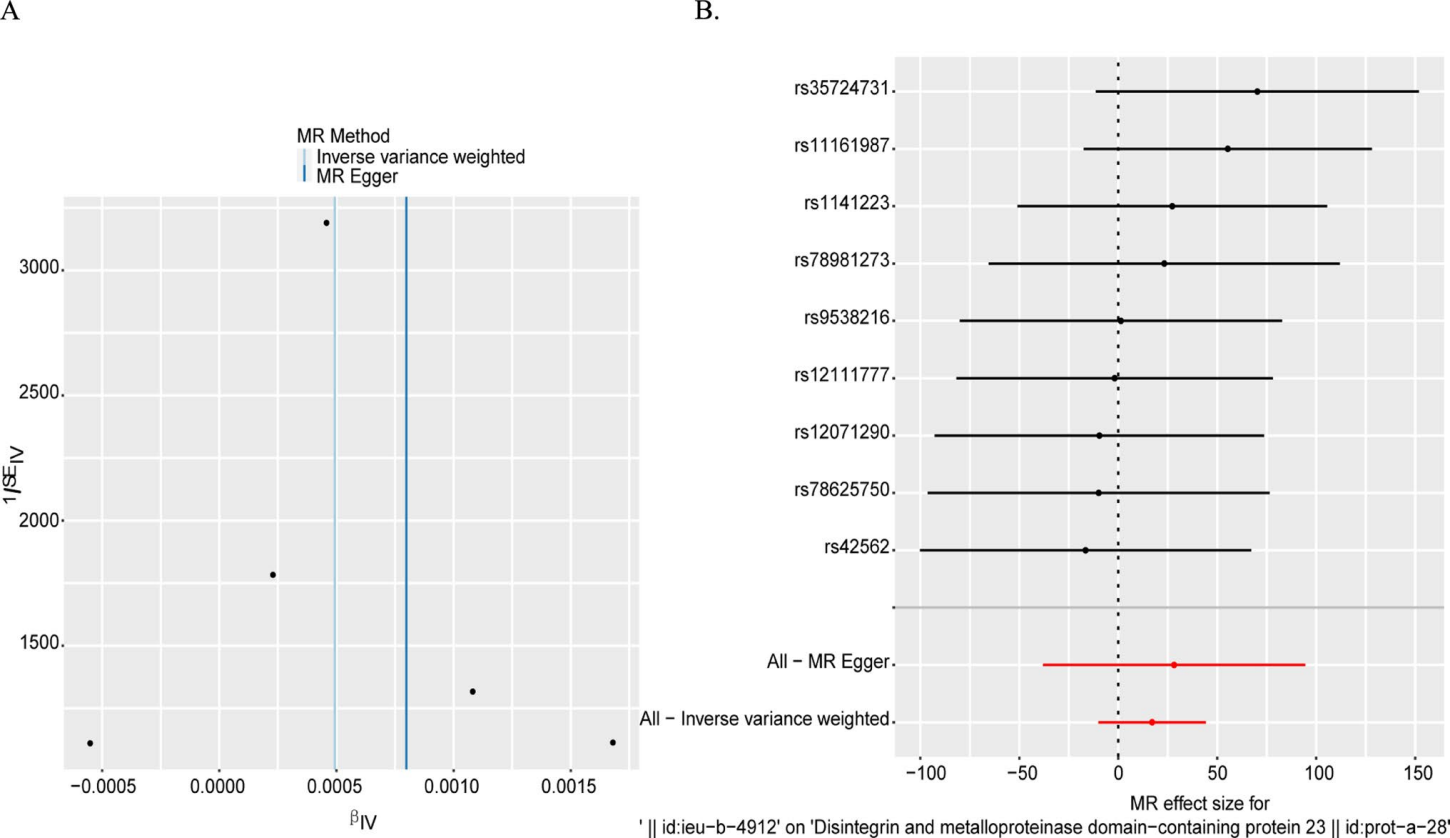
Funnel plot of heterogeneity test for Mendelian randomization analysis of blood protein indicators and head and neck cancer. **A** Funnel plot of causal effect estimation of A Disintegrin and metalloproteinase domain-containing protein 23 and head and neck cancer for each instrumental variable shows that the causal effect estimates for the Inverse variance weighted and MR Egger models are annotated with straight lines on the plots. **B** Forest plot of multiple Mendelian randomization model analysis results of causal association between the risk of head and neck cancer and blood proteome indicators

The heterogeneity of the significant results was tested using the Cochran Q test and I^2^ statistic, as shown in Table 2. The results showed that the MR Results of blood proteome indicators for head and neck cancer (Cochran Q p-value > 0.05, I^2^ < 50%) did not demonstrate considerable heterogeneity. The funnel plot of instrumental variables of ADAM23 (Fig. 3A) showed that the scatter points of causal association effects were basically symmetrically distributed, indicating that there was no potential bias in the results. MR Egger, Weighted median, Inverse variance weighted (IVW), Simple mode (SM) and Weighted mode models were used for reverse causality MR Analysis, respectively. The results were shown in a forest map (Fig. 3B).

**Table 2:** Mendelian randomization analysis heterogeneity test for the association between blood protein trait and head and neck cancer. Q: Cochran's Q test statistic. Q df: degrees of freedom for the Q test. I2 statistic reflects the proportion of heterogeneity attributed to instrumental variables in the total variability

Exposure	Method	Q	Q_df	Q_pval	I^2^ (%)
A Disintegrin and metalloproteinase domain-containing protein 23 | | id: prot-a-28	MR Egger	3.896909774	3	0.272813342	23.02%
A Disintegrin and metalloproteinase domain—containing protein 23 | | id: prot-a-28	Inverse variance weighted	3.928264879	4	0.415801186	0

Table 2 presents the results of the heterogeneity test in the MR analysis of the causal association between blood plasma protein indicators and head and neck cancer. We used Cochran’s Q test and the I^2^ statistic to assess and quantify the heterogeneity among the SNP effect estimates. For the protein indicator A Disintegrin and metalloproteinase domain-containing protein 23 (ADAM23), when using the MR Egger method, the Q value of Cochran's Q test was 3.897 with 3 degrees of freedom and a P-value of 0.273, indicating no significant heterogeneity. Simultaneously, the I^2^ statistic was 23.02%, suggesting that only 23.02% of the total variability was due to heterogeneity in the instrumental variables, which is a relatively low degree of heterogeneity. When using the Inverse variance weighted (IVW) method, the Q value of Cochran’s Q test was 3.928 with 4 degrees of freedom and a P-value of 0.416, again revealing no significant heterogeneity. The corresponding I^2^ statistic was 0, further confirming that heterogeneity was negligible. Combining the heterogeneity test results from these two MR methods, we can draw a consistent conclusion that the causal effect estimates from the selected instrumental variables did not differ substantially, supporting the reliability of the MR analysis results.

MR-Egger regression was used to test the horizontal pleiotropy of instrumental variables. The statistical hypothesis test P values of the intercept terms of each index were greater than 0.05, and the intercept was close to 0, indicating that the causal inference in this study was not affected by the horizontal pleiotropy, as shown in Table 3.

**Table 3:** Mendelian randomization analysis of horizontal pleiotropy for the association between blood protein trait and head and neck cancer

Exposure	MR-Egger intercept	Standard error	P value
A Disintegrin and metalloproteinase domain—containing protein 23 | | id: prot-a-28	0.0001264688	0.000814008	0.8863982

### Multivariate MR analysis

Multivariable MR Analyses were performed to assess the direct effects of significant blood proteome markers on head and neck squamous cell carcinoma (HNSCC) risk by adding exposure to obesity, diabetes, and smoking, respectively. Model 1 adjusted for the indirect effect of obesity, and the results showed that the significant blood protein index prot-a-28 still had a significant direct effect on HNSCC. After adjusting for the indirect effect of diabetes in model 2, prot-a-28 still had a significant direct effect on HNSCC risk. In model 3, after adjusting for the indirect effect of smoking, prot-a-28 still had significant direct effect on HNSCC (Table 4). Table 5 shows the results of the sensitivity analysis, which used leave-one-out methods to assess the robustness of the causal effect estimate for ADAM23. When all SNPs were included, the overall effect estimate was 0.000493 (p = 0.0387). These findings support the stability and reliability of the MR results for ADAM23.

**Table 4:** The results of multivariable Mendelian randomization analysis on the impact of blood protein trait and head and neck cancer. Model 1: Multivariable MR analysis of blood protein trait and obesity on head and neck cancer. Model 2: Multivariable MR analysis of blood protein trait and diabetes mellitus on head and neck cancer. Model 3: Multivariable MR analysis of blood protein trait and smoke on head and neck cancer. MR: Mendelian randomization. SNPs: Single Nucleotide Polymorphisms

Model	Exposure	Number of SNPs	OR (95%CI)	P value
Model 1	A Disintegrin and metalloproteinase domain-containing protein 23 | | id: prot-a-28	4	1.000546245	0.019544591
Model 2	A Disintegrin and metalloproteinase domain-containing protein 23 | | id: prot-a-28	3	1.000595854	8.09 e-05
Model 3	A Disintegrin and metalloproteinase domain-containing protein 23 | | id: prot-a-28	4	1.000546245	0.019544591

**Table 5:** MR leave-one-out analysis results. SNPs: Single Nucleotide Polymorphisms

Exposure	SNPs	b	Se	p
Disintegrin and metalloproteinase domain-containing protein 23 || id:prot-a-28	rs11244049	0.000572206	0.000247493	0.020776878
Disintegrin and metalloproteinase domain-containing protein 23 || id:prot-a-28	rs114865519	0.000428822	0.000262111	0.101833035
Disintegrin and metalloproteinase domain-containing protein 23 || id:prot-a-28	rs191343078	0.000403187	0.000247543	0.103365698
Disintegrin and metalloproteinase domain-containing protein 23 || id:prot-a-28	rs1921673	0.000542438	0.000419423	0.195908815
Disintegrin and metalloproteinase domain-containing protein 23 || id:prot-a-28	rs74584500	0.000551813	0.000291159	0.058061796
Disintegrin and metalloproteinase domain-containing protein 23 || id:prot-a-28	All	0.000493322	0.000238649	0.0387208

### The expression pattern, diagnostic value, and prognostic value of ADADM23 in HNSC

**Fig. 4:**
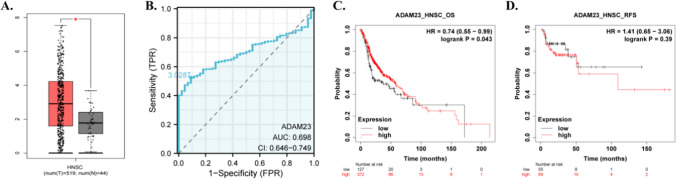
The expression, diagnostic value, and prognostic significance of ADAM23 in HNSC. **A** The box plot showing the expression levels of ADAM23 in HNSC tumor tissues (n = 519) and normal tissues (n = 44) from the TCGA and GTEx databases. **B** ROC curve analysis evaluating the diagnostic value of ADAM23 expression in distinguishing HNSC tumor tissues from normal tissues. **C** Kaplan–Meier curve comparing the overall survival (OS) of HNSC patients with high and low ADAM23 expression levels. **D** Kaplan–Meier curve comparing the recurrence-free survival (RFS) of HNSC patients with high and low ADAM23 expression levels

Figure 4A showed that the expression of ADAM23 was significantly upregulated in HNSC tumor tissues compared to adjacent normal tissues and GTEx normal tissues. Receiver operating characteristic (ROC) curve analysis demonstrated that ADAM23 expression levels could effectively distinguish between HNSC tumor tissues and normal tissues, with an area under the curve (AUC) value of 0.698 (95% confidence interval: 0.646–0.749), suggesting the potential diagnostic value of ADAM23 (Fig. 4B). Furthermore, we evaluated the relationship between ADAM23 expression levels and the prognosis of HNSC patients. Kaplan–Meier survival analysis revealed that the overall survival (OS) of the ADAM23 high-expression group was significantly better than that of the low-expression group (HR = 0.74, 95%CI 0.55–0.99, Log-rank P = 0.043) (Fig. 4C). However, there was no significant correlation between ADAM23 expression levels and recurrence-free survival (RFS) (HR = 1.41, 95%CI 0.65–3.06, Log-rank P = 0.39) (Fig. 4D).

## Discussion

In the present study, we applied two-sample Mendelian randomization to causally infer the role of the blood proteome on the risk of head and neck cancer using data from 1927 genetic associations with 1,478 blood proteins. SNPs associated with blood proteome indicators were included as instrumental variables and ADAM23 was found to be a significant instrumental variable. Causal effects estimates further indicated a significant causal association between ADAM23 and head and neck cancer. Furthermore, sensitivity analysis showed no significant heterogeneity, and MR-Egger regression analysis showed causal inference in this study was not affected by the horizontal pleiotropy. Multivariable analysis conducted to control for potential confounding by obesity, smoking, diabetes indicated this causal inference was valid. Together, these results highlighted the causal role of ADAM23 in head and neck cancer.

A Disintegrin and metalloprotease proteins (ADAMS) have been implicated to play important roles in tumor growth and metastasis [34]. ADAMS are primarily involved in the process of ectodomain shedding by acting as ectodomain sheddases, a post-translational modification [35]. ADAMS substrates are either membrane or extracellular matrix proteins and therefore they are implicated in cell adhesion and cell–matrix interactions [36]. A number of ADAM family proteins have been found expressed in various cancers and are implicated in integrin and growth factor regulation, controlling in turn, cell growth and regulation [37] Specifically, ADAM-23 is implicated in cell adhesion and has been reported in several cancers including brain [38], breast [39], gastric [40], colorectal [41] and ovarian [42] cancers. The hypermethylation of ADAM-23 has been associated with more advanced cancer of the larynx [43]. ADAM23 exhibits a tumor suppressor role and was found to promote tumor cell death and reduce cell proliferation by promotion of ferroptosis in esophageal cancer [44]. ADAM23 silencing plays a key role in tumor metastasis and progression by downregulating αvβ3 integrin activation [45]. In a pan-cancer study, ADAMs were found to be differentially expressed in tumor tissues, associated with prognosis, tumor immune scores and drug sensitivity of tumors [46]. Considering its role in αvβ3 integrin activation, ADAM23 may be a potential biomarker for anti-integrin therapies in head and neck cancer, which have been a recent focus area of research [47]. αvβ3 integrin activation plays a significant role in the progression of head and neck cancer. When activated, αvβ3 integrin promotes tumor cell migration, invasion, and metastasis of HNSC by facilitating cell adhesion to the extracellular matrix and transmitting signals that regulate cytoskeletal reorganization and matrix metalloproteinase (MMP) expression [48]. Furthermore, αvβ3 integrin activation enhances tumor angiogenesis in HNSCC by interacting with vascular endothelial growth factor receptor 2 (VEGFR2) and promoting endothelial cell survival and migration, thus contributing to tumor growth and metastatic spread [49]. Among the associated SNPs, rs11244049-T has been mapped to platelet count [19]. Platelets can promote tumor angiogenesis in head and neck cancer by releasing pro-angiogenic factors such as VEGF, PDGF, and TGF-β. By enhancing tumor vascularization, platelets provide oxygen and nutrients to support tumor growth and create a favorable microenvironment for tumor progression [50]. As a result, higher platelet counts may influence the development and progression of head and neck cancer through their effects on tumor angiogenesis [51]. In addition, rs1921673 has been mapped to ADAM23 expression [22]; however, there is currently no literature directly linking this SNP to the risk of head and neck cancer. Further functional studies are needed to elucidate the precise mechanisms by which these SNPs contribute to head and neck cancer susceptibility and to assess their impact on protein function, prognosis, and potential for guiding targeted therapy.

Although our study results support a causal relationship between ADAM23 and head and neck cancer, we cannot currently determine the exact direction of causality. One possible explanation is that elevated ADAM23 protein levels lead to an increased risk of head and neck cancer. However, we cannot rule out the alternative possibility that ADAM23 gene polymorphisms affect head and neck cancer risk by altering protein levels. Furthermore, we need to consider the potential for reverse causality or bidirectional effects. The occurrence and development of head and neck cancer may, in turn, influence the expression levels of ADAM23, or there may be a bidirectional relationship with mutual influence between ADAM23 and head and neck cancer. To further elucidate the causal direction, future studies should integrate genotype, proteomic, and clinical data and employ more sophisticated causal inference methods, such as bidirectional Mendelian randomization analysis. Additionally, functional experiments are needed to validate the specific mechanisms of action of ADAM23 in the development of head and neck cancer.

The identification of ADAM23 as a causal risk factor for head and neck cancer has important implications for risk stratification, early detection, and precision prevention strategies. Circulating ADAM23 levels could potentially be incorporated into risk prediction models to improve their performance and guide personalized screening and surveillance approaches. Moreover, understanding the biological pathways through which ADAM23 influences head and neck cancer development may inform the development of targeted interventions to mitigate its harmful effects. Our findings also emphasize the value of integrating genetic and proteomic data to uncover novel disease mechanisms and druggable targets. As large-scale proteomic profiling becomes increasingly feasible, our study provides a framework for future investigations of the plasma proteome's role in cancer etiology.

The advantages and limitations of MR research in tumor susceptibility studies needs to be mentioned. MR research utilizes genetic variations as instrumental variables, which can avoid the interference of confounding factors and reverse causality in traditional observational studies to a certain extent, thus more accurately inferring the causal relationship between exposure factors and outcomes. This methodological advantage has led to its increasing application in the field of tumor susceptibility research. In particular, applying the latest SNP-protein association data to perform MR analysis can directly reveal the pathogenic mechanisms of tumors at the protein level, which has important guiding significance for drug development. However, MR research also has some inherent limitations, such as endogeneity assumptions and genetic pleiotropy. In future studies applying the MR method, it is necessary to more comprehensively collect genetic instrumental variables, strengthen pleiotropy testing and sensitivity analysis, and, when necessary, use other causal inference methods for complementary verification to improve the reliability of the research results.

The limitation of the current research is noteworthy to be highlighted. Firstly, it is important to note that while this study utilized a substantial dataset of 1,478 plasma proteins, it does not represent the entirety of the human plasma proteome, which is estimated to comprise thousands of proteins. The use of the latest proteomic technologies could enable the interrogation of a more comprehensive set of proteins in future studies.By expanding the scope of analyzed proteins, we may uncover additional causal associations between plasma proteins and head and neck cancer risk. Secondly, it is important to acknowledge that the predominantly European ancestry of our study population may limit the generalizability of the findings. Differences in genetic background, environmental factors, and lifestyle habits across ethnic groups can lead to heterogeneity in genotype–phenotype associations and modulate gene expression and disease risk. To extend the applicability of our results, future research should consider conducting validation studies in diverse ethnic populations, performing population-specific Mendelian randomization analyses, incorporating ethnicity interaction terms in the analyses, and integrating multi-omics data for cross-population investigations. Furthermore, we must acknowledge another limitation of our study. Although Mendelian randomization has certain advantages in reducing confounding and reverse causality, as an observational study, we cannot completely exclude all possible confounding factors, especially some unmeasured or unknown confounders. Moreover, our results may be influenced by the pleiotropy of genetic variants. To further validate our findings and overcome the limitations of observational studies, we strongly recommend conducting more prospective studies, such as cohort studies or clinical trials, in the future research. Prospective studies can better control for confounding factors, provide more direct evidence of causal relationships, and assess the temporal relationship between exposure and outcome.

To build upon the findings of this study, several experimental directions should be pursued. First, prospective studies are needed to validate the association between pre-diagnostic ADAM23 levels and head and neck cancer risk and evaluate its potential as an early detection biomarker. Second, functional studies are warranted to elucidate the biological mechanisms by which ADAM23 contributes to head and neck carcinogenesis, including its effects on cell adhesion, proliferation, and invasiveness. The molecular mechanisms regulating ADAM23 expression, such as the role of epigenetic alterations like promoter methylation, merit further study. Clarifying the mechanism of action of ADAM23 will help discover new drug intervention targets. For example, small molecule compounds, antibodies, or peptide drugs that specifically inhibit ADAM23 expression or activity can be developed, and therapeutic strategies targeting downstream signaling pathways of ADAM23 can also be designed. ADAM23 may also be a potential molecular biomarker for screening and evaluating the efficacy of head and neck cancer drugs. Third, the therapeutic potential of targeting ADAM23 should be explored using in vitro and in vivo models. Additionally, given the genetic architecture of head and neck cancer, it will be important to investigate whether the causal role of ADAM23 varies across molecular subtypes or is modified by environmental exposures. Furthermore, this study suggests that elevated plasma ADAM23 levels may be a risk factor for the occurrence of head and neck cancer, providing an important direction for the development of plasma protein biomarkers for head and neck cancer. Subsequent studies can further validate the correlation between ADAM23 levels and the risk of head and neck cancer in large sample cohorts and evaluate its potential as an early screening and diagnostic biomarker. Meanwhile, other plasma protein indicators related to ADAM23 are also worthy of attention, and there is hope to construct a combination model of protein biomarkers to improve the sensitivity and specificity of head and neck cancer risk prediction. In addition, dynamically monitoring changes in ADAM23 levels may help evaluate the efficacy and prognosis of head and neck cancer. Addressing these questions will be crucial for translating our findings into clinical applications and advancing our understanding of head and neck cancer biology.

## Limitation

- While this study utilized GWAS summary statistics from large publicly available datasets, the sample size for head and neck cancer cases was relatively modest. Future studies with larger head and neck cancer sample sizes are needed to validate and extend these findings.
- While the multivariable analysis adjusted for major risk factors like obesity, diabetes and smoking, residual confounding from unmeasured factors cannot be completely ruled out. Future studies incorporating a wider range of covariates would strengthen causal inference.
- The blood protein GWAS data included 1478 proteins. While this represents a sizable proportion of the blood proteome, future studies capturing a more comprehensive set of proteins using latest proteomic technologies could potentially uncover additional causal proteins.
- The SNPs studied as instrumental variables may have pleiotropic effects beyond the specific blood proteins considered here. More extensive sensitivity analysis and functional validation of the SNPs would further clarify their biological effects.
- This study provides evidence for the causal effect of the specific protein ADAM23. However, the biological mechanisms linking ADAM23 to head and neck cancer pathogenesis warrant further investigation through molecular and cell biology experiments.
- The study population was primarily of European ancestry. The generalizability of the findings to other ethnic groups needs to be established through additional studies.

## Conclusion

Taken together, the present study applied two-sample MR and highlighted a significant causal role of genetically dysregulated ADAM23 with head and neck cancer risk. The strengths of our study include the study of a large number of blood protein traits, large sample sizes, and sensitivity analysis and multivariate analysis that decrease the possibility of bias. Future studies are warranted to address the causal mechanisms underlying the role of ADAM23 in mediating head and neck cancers, and its role as a potential therapeutic target and biomarker.

## Data Availability

All datasets utilized in the study are publicly available.

## Code availability

The codes during the current study are available from the corresponding author on reasonable request.
